# Glioblastoma with cerebrospinal fluid dissemination and MYC gene amplification: A transitional or atypical glioblastoma with primitive neuronal components

**DOI:** 10.1097/MD.0000000000044072

**Published:** 2025-11-14

**Authors:** Wenhui Zhang, Lihao Lin, Yongxue Li, Yan Wang, Haoyu Shen, Yi Guan

**Affiliations:** a Department of Neurosurgery, The First Hospital of Jilin University, Changchun, China; b Department of Neurosurgery, The Second Hospital of Tianjin Medical University, Tianjin, China.

**Keywords:** cerebrospinal fluid, extracranial metastasis, glioblastoma, MYC gene, primitive neuronal components

## Abstract

**Rationale:**

Glioblastoma (GBM) is the most common primary malignant intracranial tumor in adults. GBMs with primitive neuronal components (GBM-PNC) represent a rare subtype of GBM, accounting for 0.5% of cases, often characterized by cerebrospinal fluid (CSF) dissemination and extracranial metastasis (ECM). Among the primitive neuronal components (PNC), MYC gene amplification is found in 43% of cases. Once diagnosed, treatment becomes extremely challenging; therefore, early identification and timely intervention are crucial.

**Patient concerns:**

A 60-year-old male presented with a 5-day history of headache and limb weakness. He had no prior neurological disorders and no family history of brain tumors.

**Diagnoses:**

Magnetic resonance imaging of the head revealed a heterogeneously enhancing mass adjacent to the right lateral ventricle. Although postoperative immunohistochemistry, MYC gene amplification on molecular testing, and disease progression were consistent with the characteristics of GBM-PNC, the absence of corresponding morphological evidence led to a diagnosis of GBM NOS, World Health Organization grade IV. Postoperative examination demonstrated tumor metastases in the head, thoracic, and lumbosacral regions, suggesting ECM spread via CSF dissemination. In combination with the pathological findings, the case was ultimately considered a transitional or atypical GBM-PNC.

**Interventions:**

After evaluating the patient’s condition, the tumor was completely resected surgically. Postoperatively, the patient underwent radiotherapy, chemotherapy, and regular follow-up. Two months later, tumor metastases were detected. In addition to the original treatment regimen, intrathecal methotrexate injections were administered.

**Outcomes:**

Two months after surgery, tumor metastasis was detected. Treatment with radiotherapy, chemotherapy, and intrathecal drug injections effectively controlled disease progression. However, the patient’s condition deteriorated 10 months postoperatively, and he ultimately passed away 14 months after the initial surgery.

**Lessons:**

Based on this case and a review of relevant literature, it can be inferred that MYC amplification may serve as an early predictive biomarker for ECM via CSF dissemination in GBM. Furthermore, MYC gene overexpression may promote the transformation of primitive cells, and this case may represent a transitional or atypical GBM-PNC. This case suggests that clinicians should remain vigilant for the potential of CSF dissemination and ECM in GBM patients, particularly when MYC amplification is present, which could serve as an early warning of distant metastasis.

## 1. Introduction

Glioblastoma (GBM) is the most common primary malignant intracranial tumor in adults, accounting for 48.6% of cases, with a poor prognosis and a median survival of only 15 months.^[1]^ Most GBMs progress locally, and extracranial metastasis (ECM) via cerebrospinal fluid (CSF) dissemination is relatively rare. Currently, no specific predictive biomarkers for this type of ECM, making early prevention and diagnosis challenging. GBMs with primitive neuronal components (PNC) are a rare subtype, first formally recognized by the World Health Organization (WHO) in 2016.^[2]^ These tumors account for approximately 0.5% of all GBM cases and are often associated with a high propensity for CSF dissemination and ECM.^[3]^ GBM-PNC exhibits both traditional astrocytic regions with high glial fibrillary acidic protein (GFAP) expression and primitive neuronal regions with low GFAP expression and high expression of neuronal markers such as synaptophysin (SYN), soluble protein-100, and neuron-specific enolase. MYC gene amplification is found in 43% of the primitive neuronal component.^[4]^

In this report, we present a case of GBM, which, despite no evidence of metastasis on preoperative PET-CT, developed CSF dissemination to the thoracic, lumbar, and sacral regions of the spinal canal 2 months post-surgery. Immunohistochemical analysis showed partial GFAP expression, a Ki-67 proliferation index of 70%, and positive synaptophysin expression, but no morphological evidence of PNC. Molecular testing revealed MYC gene amplification. We suspect this may represent a transitional or atypical GBM-PNC, with MYC amplification serving as a significant indicator for CSF dissemination-related ECM, a finding not previously reported. This case highlights the existence of this unique type of GBM-PNC and, through a literature review, suggests that MYC amplification could serve as an early predictive biomarker for CSF dissemination, offering valuable insights for early prevention and management of ECM in GBM.

## 2. Case presentation

### 2.1. Preoperative examination

**Figure 1.**
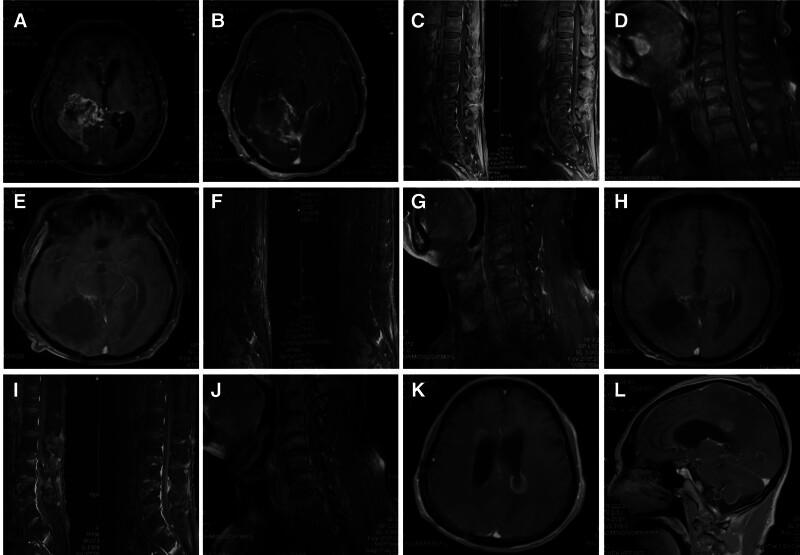
Imaging. (A) Preoperative head MRI shows an irregularly enhanced signal measuring 7.07 cm × 5.04 cm × 4.47 cm in the right hippocampus, temporal–occipital lobe, thalamus, corona radiata, and the splenium of the corpus callosum, invading the right lateral ventricle. (B) Postoperative 1-month head MRI demonstrates complete resection of the tumor. (C) Postoperative 2-month lumbar MRI reveals small nodular abnormal enhancement at the L2–3, L4–5 intervertebral disc levels and S2 vertebral body level in the spinal canal. (D) Cervicothoracic MRI shows a significant enhancing nodular signal in the spinal canal at the T2 vertebral body level. (E) Postoperative 5-month head MRI shows a reduction in both the original abnormal signal and surrounding enhanced signal. (F) Postoperative 5-month lumbar MRI shows no significant change in the abnormal enhancement signal within the lumbar spinal canal. (G) Postoperative 5-month cervicothoracic MRI shows a reduction in the abnormal enhancement signal in the spinal canal at the T2 vertebral body level. (H) Postoperative 8-month head MRI shows further reduction in the intensity and extent of the original abnormal signal. (I, J) Postoperative 8-month cervicothoracic MRI reveals no significant enlargement of the original abnormal signal in the spinal canal. (K, L) Postoperative 10-month head MRI shows abnormal enhancement signals in multiple areas of the cerebral cortex and periventricular regions consistent with tumor presence.

A 60-year-old male patient was admitted with a 5-day history of headache and limb weakness. He had no prior neurological disorders and no family history of brain tumors. Head magnetic resonance imaging (MRI) revealed an abnormal signal measuring 7.07 × 5.04 × 4.47 cm in the right hippocampus, temporal–occipital lobe, thalamus, corona radiata, and corpus callosum, with invasion into the right lateral ventricle. After gadolinium injection, the tumor showed heterogeneous enhancement, with surrounding patchy edema (Fig. 1A), initially suspected to be malignant glioblastoma. The patient underwent PET–CT (examination no. 20206502) and lumbar MRI (examination no. P01235994), both of which did not show metastatic lesions.

### 2.2. Surgery

**Figure 2.**
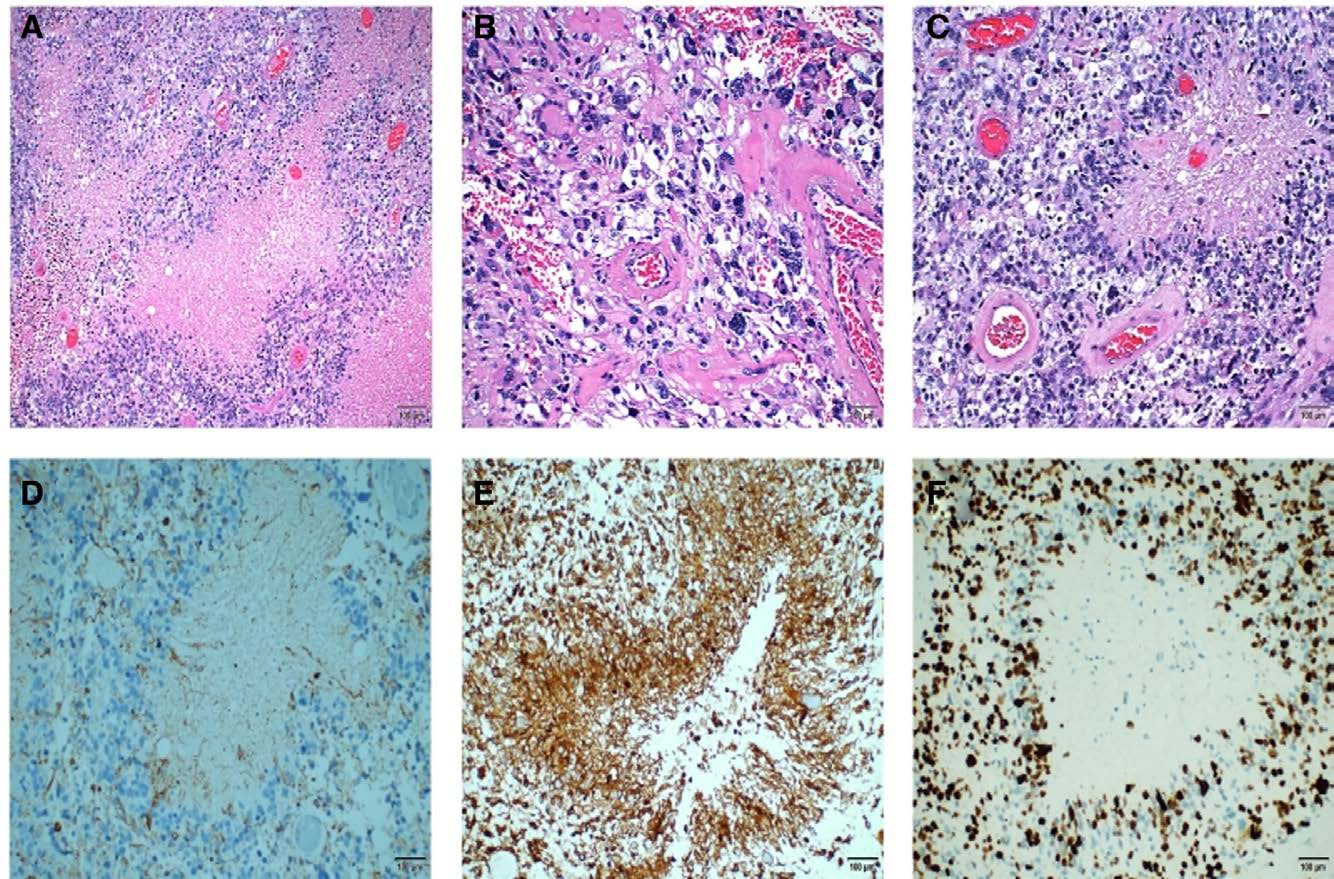
Pathology. (A–C) Histological examination reveals typical astrocytic cells with irregular necrotic areas, surrounded by tumor cells arranged in a palisade pattern. (D–F) Immunohistochemistry, (D) GFAP expression is partially positive and partially negative. (E) SYN expression is positive. (F) Ki-67 proliferation index is 70%. GFAP = glial fibrillary acidic protein, SYN = synaptophysin.

**Figure 3.**
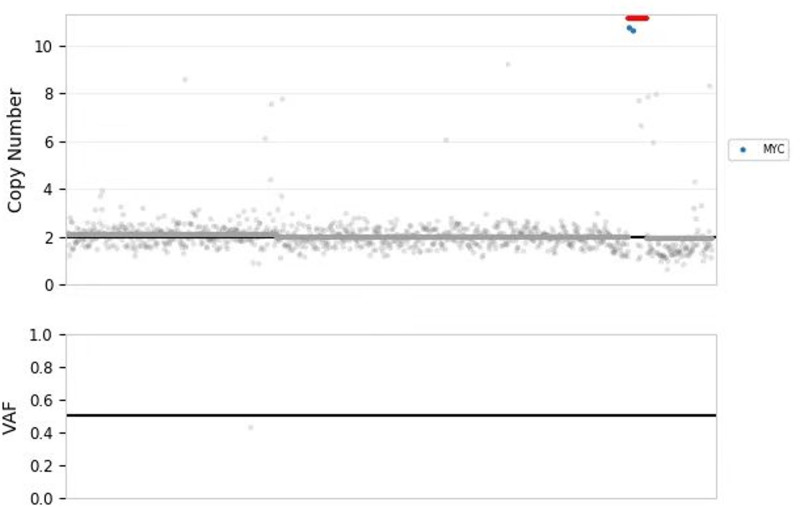
Molecular testing. MYC gene amplification with a copy number of 14.33.

After evaluating the patient’s condition, the tumor was surgically removed completely. Postoperative pathology confirmed glioblastoma NOS, WHO grade IV. Pathological morphology revealed typical astrocytic cells and irregular necrotic areas, with tumor cells arranged in a fence-like distribution (Fig. 2A–C). Immunohistochemistry showed partial GFAP expression (Fig. 2D), synaptophysin positivity (Fig. 2E), Ki-67 proliferation index of 70% (Fig. 2F), tumor protein 53 expression (70%), alpha thalassemia/mental retardation syndrome X-linked positivity, oligodendrocyte transcription factor 2 positivity, and negative mutation for isocitrate dehydrogenase 1 R132H. Molecular testing revealed MYC gene amplification with a copy number of 14.33 (Fig. 3).

### 2.3. Post-operation follow-up

One month post-surgery, a head MRI was performed, confirming complete tumor resection (Fig. 1B), and the patient had a good recovery. Radiation therapy and temozolomide chemotherapy were subsequently initiated.

Two months post-surgery, the patient presented with lumbar pain, and lumbar MRI showed small nodular abnormal enhancement at the L2–3, L4–5 intervertebral disc levels and S2 vertebral canal, approximately 0.3 to 0.4 cm (Fig. 1C), suggestive of metastatic lesions. To rule out cervical and thoracic tumor metastasis, MRI of the cervical and thoracic spine was performed, revealing a nodular abnormal signal with significant enhancement at the T2 vertebral level, approximately 0.5 × 0.6 × 0.8 cm, suggestive of thoracic spinal canal metastasis (Fig. 1D). To exclude metastasis at other sites, PET-CT was performed, which showed slightly elevated metabolic changes near the surgical field at the right occipital lobe margin, near the falx cerebri, corpus callosum, occipital lobe, and at the T2 vertebral and T11 to T12 spinal canal levels (examination no. P20121128), suggestive of metastasis. Based on the patient’s medical history and current condition, CSF dissemination was considered, and combining immunohistochemistry findings, the intracranial GBM was considered to be a GBM-PNC. However, due to the absence of PNC in the surgical pathology, a definitive diagnosis could not be made. Upon discovering metastasis, the patient and family opted for conservative treatment, and the treatment plan was adjusted, including intrathecal methotrexate injection in addition to radiation and chemotherapy, with oral temozolomide administered periodically after discharge.

Five months post-surgery, follow-up MRI showed a reduction in the original abnormal signal in the head (Fig. 1E), with no significant change in the abnormal signal in the lumbar spinal canal (Fig. 1F). The abnormal signal at the T2 vertebral level also showed a reduction (Fig. 1G). Eight months post-surgery, follow-up MRI showed further reduction in the abnormal signal intensity and extent in the head (Fig. 1H), while abnormalities in the thoracic-lumbar spine remained largely unchanged (Fig. 1I, J). PET-CT showed a reduction in the previously high metabolic brain lesions, and no high metabolic lesions were observed in the thoracic-lumbar spine, suggesting suppressed tumor activity (examination no. P21061610). Due to the favorable tumor imaging response and the potential for serious complications and drug resistance with long-term chemotherapy, the radiation oncology team recommended temporarily halting temozolomide treatment.

Ten months post-surgery, the patient presented to a local hospital with difficulty swallowing due to tumor recurrence (Fig. 1K, L). The patient and family chose conservative symptomatic treatment and did not undergo surgery.

Fourteen months post-surgery, the patient’s condition worsened, and he died due to respiratory and circulatory failure.

## 3. Clinical course

**Figure 4.**
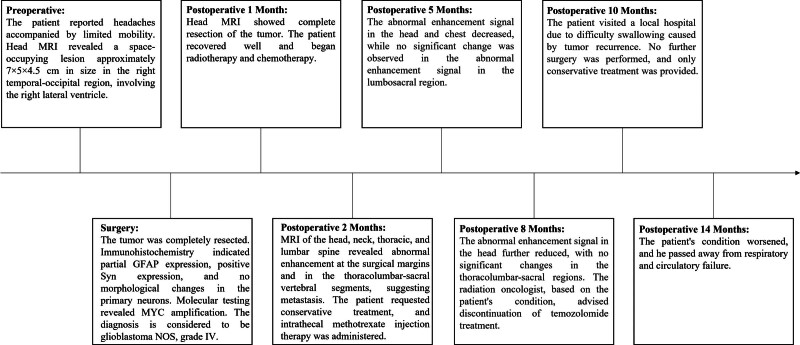
Timeline.

Figure 4 illustrates the patient’s clinical progression.

## 4. Discussion

GBM is the most common primary malignant intracranial tumor in adults, with a median survival time of only 15 months.^[1]^ It commonly recurs locally, and ECM through CSF dissemination typically occurs in the later stages of the disease. In 1931, Cairns et al reported the first case of GBM that could metastasize to the spinal cord along the CSF pathway.^[5]^ Literature suggests that the probability of malignant glioma exhibiting CSF dissemination ranges from 6% to 60%, but the incidence of symptomatic spinal metastasis is only 1.3% to 8.8%, making it relatively uncommon.^[6]^ Mechanistically, tumor invasion of the choroid plexus in the ventricles plays an important role in the dissemination of the tumor through CSF during ECM.^[7]^ However, there are currently no specific predictive biomarkers for this type of metastasis, posing a significant challenge for the early prevention and detection of GBM-related ECM.

### 4.1. MYC genes

MYC, one of the most widely studied oncogenes, is frequently dysregulated in various cancers in a tissue-specific manner. The MYC oncogene family consists of 3 members: C-MYC, N-MYC, and L-MYC. MYC amplification has been significantly associated with the progression of gliomas.^[8–10]^ N-MYC amplification has been observed in 42.9% of GBM cases.^[11]^ Furthermore, MYC genes play a critical role in promoting tumor metastasis. David R. Ghasemi et al reported a special subtype of ependymoma characterized by MYCN amplification, named SP-EPN-MYCN. In this subtype, the tumor often undergoes diffuse leptomeningeal dissemination throughout the central nervous system, resulting in invasive growth along the spinal cord.^[12]^ Additionally, C-MYC amplification has been significantly associated with metastatic medulloblastoma in children.^[13–15]^ Moreover, C-MYC promotes the generation of mesenchymal cells with high migratory ability through targeting epithelial–mesenchymal transition (EMT). In liver cancer, MYC can influence metastasis in both EMT-dependent and -independent ways.^[16,17]^ In glioma cells, C-MYC also enhances its stimulatory effects on EMT by inducing the Wnt/β-catenin pathway.^[18]^ In this case, the tumor’s invasion of the choroid plexus caused the tumor cells to disseminate through the CSF to ECM. MYC amplification may have accelerated this process. All of the above suggest that when a tumor is located in areas prone to choroid plexus invasion and there is an increase in MYC copy number, clinicians should be highly vigilant for the occurrence of tumor ECM, and focus on early prevention and diagnosis.

### 4.2. Glioblastoma with primitive neuronal components

GBM-PNC was explicitly classified in the 2016 WHO classification of central nervous system tumors,^[2]^ and this subtype is extremely rare. Regarding the diagnosis of GBM-PNC, Arie Perry et al proposed that only gliomas with compelling primitive neuronal cell characteristics, both morphologically and immunohistochemically, should be diagnosed as GBM-PNC.^[4]^ Histopathologically, GBM-PNC consists of 2 components: one is the classic astrocytic tumor area of GBM, which expresses GFAP and Vimentin but does not express SYN; the other is a clearly defined nodular primitive cell area, which exhibits neuronal differentiation (e.g., Homer Wright rosettes). This area lacks GFAP expression but is typically SYN-positive and may occasionally show MYC or MYCN amplification.^[4]^

In this case, the tumor presented with typical astrocytic cells and irregular necrotic areas, with surrounding tumor cells arranged in a palisading pattern. Despite repeated observation, no primitive neuronal cell morphology was observed. The immunohistochemical profile displayed some primitive neuronal characteristics, including SYN positivity, absence of GFAP expression, and a significantly elevated Ki-67 proliferation index (+70%), with molecular testing indicating MYC gene amplification. Although the immunohistochemical and genetic results, as well as the disease progression, are consistent with the features of GBM-PNC, the absence of related morphological evidence led to a final pathological diagnosis of glioblastoma NOS, WHO grade IV. Based on these observations, we propose 2 hypotheses:

First, we speculate that the tumor in this case is in a dynamic developmental stage, with certain GBM cells differentiating into primitive neuronal cells. There are 2 main hypotheses regarding the origin of PNC in GBM-PNC: one is that the PNC components arise from preexisting gliomas, most commonly in secondary GBM.^[4]^ It has been reported that 25% of GBM-PNC patients have a clear history of low-grade glioma.^[19]^ The second hypothesis is that the PNC components are the result of clonal proliferation of tumor stem cells or progenitor cells.^[4,20]^ In vitro experiments have demonstrated that overexpression of C-MYC protein can convert GFAP-positive astrocytes into Nestin-positive, GFAP-negative primitive neuronal cells.^[21]^ Therefore, we hypothesize that in this patient, on top of forming GBM, further genetic mutations occurred in the relevant cells, particularly MYC mutations and amplifications, which resulted in the positive primitive neuronal characteristics observed at the immunohistochemical and molecular levels. However, this process is in its early stages and has not yet produced morphological changes, which is why no related bidirectional histological features were observed under the microscope. Perhaps, if the patient had delayed medical consultation or experienced recurrence and metastasis during the disease course, relevant morphological features would have been observed, leading to a definitive diagnosis of GBM-PNC.

The second hypothesis is that this tumor represents an atypical form of GBM-PNC without classic morphological features. This subtype lacks the classic morphological characteristics of GBM-PNC but exhibits similar features in terms of immunohistochemistry, molecular changes, and disease progression. However, the validity of this hypothesis requires further case studies for confirmation.

Given these 2 hypotheses, we believe that the incidence of GBM-PNC may be far higher than currently recognized, as some tumors with immunohistochemical characteristics of PNC are diagnosed as GBM due to the lack of morphological evidence under the microscope. This highlights the highly heterogeneous nature of GBM, and it is extremely challenging to classify GBM solely based on microscopic, macroscopic, or imaging features.^[22]^ Molecular-level testing is increasingly important in the diagnosis and treatment of GBM.

### 4.3. Connection and enlightenment

GBM-PNC often presents with CSF dissemination and has a high tendency for spinal metastasis, with metastasis rates reaching up to 40%.^[3,4,10]^ Related studies have indicated that the PNC of GBM-PNC are primarily responsible for metastasis.^[23,24]^ In primitive neurons, N-MYC or C-MYC gene amplification accounts for 43%.^[4]^ As mentioned above, overexpression of C-MYC can promote the transformation of primitive cells, leading to metastasis.^[21]^ In this case, the preoperative spinal MRI and PET-CT showed no significant abnormalities, but nodular metastatic lesions were discovered 2 months postoperatively. We suspect that the molecular changes in this patient may have triggered CSF dissemination.

Given that the patient later developed significant CSF dissemination, we administered whole spinal irradiation combined with intrathecal methotrexate injection, resulting in a reduction of abnormal lesions. Therefore, we believe that regardless of whether a patient is diagnosed with GBM-PNC, if pathological examination of the tumor tissue reveals high Ki-67 expression and absence or reduced expression of GFAP, additional genetic testing should be promptly performed, and the risk of CSF dissemination should be closely monitored to ensure early prevention and treatment. However, if the patient is diagnosed with GBM-PNC, in addition to the treatments mentioned above, platinum-based chemotherapy should also be considered to improve prognosis and survival.^[4]^

## 5. Conclusions

GBM is the most common malignant tumor of the central nervous system. When the tumor is located near the ventricles, there is a risk of choroid plexus invasion, and if molecular testing indicates MYC gene amplification, we must be highly vigilant for the occurrence of tumor ECM and take early preventive and therapeutic measures. Furthermore, when the tumor exhibits both GBM cell characteristics and primitive neuronal cell features on immunohistochemistry, even in the absence of typical PNC morphological characteristics, the possibility of GBM-PNC should still be considered. This could represent a transitional or atypical form, and thus we should be aware of the clinical features of CSF dissemination in this type of tumor and promptly adjust the treatment plan.

## Acknowledgments

The authors would like to sincerely thank the patient for her participation in this study.

## Author contributions

**Conceptualization:** Wenhui Zhang, Yi Guan.

**Data curation:** Yongxue Li.

**Formal analysis:** Yan Wang, Yi Guan.

**Funding acquisition:** Yi Guan.

**Investigation:** Lihao Lin, Yan Wang.

**Methodology:** Lihao Lin, Haoyu Shen.

**Project administration:** Lihao Lin.

**Resources:** Yi Guan.

**Supervision:** Lihao Lin, Yi Guan.

**Software:** Yongxue Li.

**Validation:** Yi Guan.

**Visualization:** Wenhui Zhang.

**Writing – original draft:** Wenhui Zhang, Yi Guan.

**Writing – review & editing:** Wenhui Zhang, Yi Guan.
